# Augmented inhibition of angiogenesis by combination of HER2 antibody chA21 and trastuzumab in human ovarian carcinoma xenograft

**DOI:** 10.1186/1757-2215-3-20

**Published:** 2010-08-19

**Authors:** Anli Zhang, Guodong Shen, Ting Zhao, Guihong Zhang, Jing Liu, Lihua Song, Wei Wei, Ling Bing, Zhengsheng Wu, Qiang Wu

**Affiliations:** 1 Department of Pathology, Anhui Medical University, Meishan Road, Hefei, China; 2 Institute of Clinical Pharmacology, Anhui Medical University, Meishan Road, Hefei, China; 3 Affiliated Anhui Provincial Hospital, Anhui Medical University, Meishan Road, Hefei, China; 4 Anhui Anke Biotechnology Co. Ltd, Haiguan Road, Hefei, China; 5 School of Life Science, University of Science and Technology of China, Huangshan Road, Hefei, China

## Abstract

**Background:**

chA21 is a novel tumor-inhibitory antibody which recognized subdomain I of HER2 extracellular domain with an epitope distinct from other HER2 antibodies. Previously, we demonstrated that chA21 inhibits human ovarian carcinoma cell line SKOV-3 growth *in vitro *and *in vivo *study. In this study, we further investigated the anti-angiogenic efficacy combination of chA21 with trastuzumab in SKOV-3 xenograft model.

**Methods:**

Nude mice were s.c. challenged with SKOV-3 cells and received treatment of chA21 alone, trastuzumab alone or both antibodies together twice a week for 21 days. Tumor volume and microvessel density (MVD) were evaluated. The effect of chA21 plus trastuzumab treament on vascular endothelial growth factor (VEGF) secretion, endothelial cells proliferation and migration, and the status of HER2 downstream pathway AKT/phosphorylated AKT (pAKT) were evaluated *in vitro*.

**Results:**

*In vivo *study combination of chA21 with trastuzumab resulted in reduce tumor growth and angiogenesis than each monotherapy. *In vitro *study, the combination of chA21 with trastuzumab inhibits VEGF secretion, endothelial cells proliferation and migration. Furthermore, the combination treatment inhibits pAKT expression.

**Conclusion:**

Our findings suggested that the combination of chA21 with trastuzumab can cause augmented inhibition of angiogenesis in SKOV-3 xenograft model. Inhibition of agniogenesis may through suppression of AKT pathway. The therapeutic benefits of combination chA21 with trastuzumab warrant further study in an attempt to make the translation into the clinic.

## Introduction

Epithelial ovarian carcinoma is the most lethal gynecologic malignancy and resulting in high mortality rates among women patients [1]. Despite the advances in surgery, chemotherapy and radiotherapy, the average time of clinical remission is 2.5 years and approximately 20% of patients never achieve remission [2]. Thus it underscores the need for new therapeutic strategies that can be translated to the clinical treatment.

HER2, also named ErbB2/p185*^her2/neu^*, is a key member of the epidermal growth factor receptor (EGFR) family. Overexpression of HER2 is associated with tumor metastasis and poor prognosis [3]. HER2 overexpression has been reported to in 15% to 30% of ovarian carcinoma patients [4,5]. HER2-targeted therapy with monoclonal antibodies (mAbs) is a promising strategy for the ovarian carcinoma, although trastuzumab (trademark: herceptin, Genetech, Roche) has not got such great success in ovarian carcinoma as in breast or gastric cancer [6,7].

Previously we have developed a new HER2 mAb A21. This new antibody is a single-chain chimeric derivatives of chA21, which recognizes a conformational epitope distinct from trastuzumab and other HER2 therapeutic antibodies, thus it may represents a novel target site for HER2 therapeutics [8-11].

It is well accepted that angiogenesis plays a key role in tumor growth and metastasis. Research has shown that HER2 signaling is invovled in angiogenesis [12,13]. HER2 antibody trastuzumab have been shown to inhibit angiogenesis in HER2-overexpressing tumor cells [14]. The HER2 phosphorylates downstream substrates and activates a variety of signaling cascades, including the phosphatidylinositol-3 kinase (PI3K)/serine/threonine-specific protein kinase (AKT), and it regulates various cell functions especially in tumor growth, and angiogenesis [15].

In a previous study, we had found chA21 monotherapy could inhibit human ovarian carcinoma cell line SKOV-3 growth *in vitro *and *in vivo *[16]. In this study, we further investigated if more effective inhibition of angiogenesis is one of the underlying causes of the better therapeutic efficacy of the chA21 with trastuzumab combination in SKOV-3 xenograft model.

## Materials and methods

### Humanized monoclonal antibodies and cell lines

HER2 antibody chA21 was prepared as described in previous study [8]. Trastuzumab was purchased from Roche company (Shanghai, China).

Human ovarian carcinoma cell line SKOV-3 and human umbilical vein endothelial cells (HUVECs) were obtained from the American Type Culture Collection. SKOV-3 cells were cultured in RPMI 1640 (Gibco, USA) supplemented with 10% fetal bovine serum (Gibco, USA). HUVECs were maintained in F-12 nutrient mixture (Invitrogen, USA) enriched with 10% new-born calf serum (Invitrogen, USA).

### Mice xenograft model

Female BALB/c nude mice at 6-8 weeks of age were purchased from Nanjing Laboratory Animal Center of China. The experimental animal study protocols were approved by the Committee for Ethics in Animal Experimentation in University of Science and Technology of China. For tumor xenograft model, mice were subcutaneously injected with 5×10^6 ^SKOV-3 cells into the left flank. After inoculation, animals were weighed and tumor sizes were measured twice a week with calipers. Tumor volumes were calculated by the formula: (smaller diameter)^2 ^× larger diameter × 0.5. When tumor volume reached about 70 mm^3^, the mice bearing xenografts were randomly assigned into four groups (n = 8): normal saline control, chA21 alone (30 mg/kg), trastuzumab alone (20 mg/kg), and chA21 plus trastuzumab (30 mg/kg + 20 mg/kg). Drug were delivered twice a week via caudal vein. All animals were killed after treatment for 21 days. The tumors were removed, weighed and fixed in 10% neutral buffered formalin for pathological study. The tumor inhibition ratio (TIR) was calculated as previous study: (1-experimental tumor mean weight/control tumor mean weight) × 100% [17].

### Immunohistochemistry examination

The sections of paraffin-embedded tissue from SKOV-3 nude mice xenografts were dewaxed and rehydrated. Immunohistochemistry procedure was performed using DAKO Envision Plus kit (DAKO) according to the manufacturer's instructions. After antigen retrieval with autoclaving in citric acid, and inactivating endogenous peroxidase with 3% H_2_O_2_, the slides were incubated with the rabbit anti-mouse antibody CD34 (1: 200, Bioss, China) or the rabbit anti-human antibody VEGF (working solution, ZhongShan, China) overnight at 4°C. Second antibody conjugated with peroxidase labeled polymer was applied for 30 min at room temperature. The sections were developed in 3,3-diaminobenzidine and counterstained with hematoxylin. As a negative control, sections were stained normal human serum instead of the primary antibody. The mean optical density (MOD) was quantitatively analyzed using Image-pro Plus 5.02 (Media Cybernetics Inc, USA) for VEGF expression. MVD was determined by counting the number of microvessels (marked by CD34 staining) per high-power field (200×) in the sections as previously described [18].

### ELISA VEGF secretion

SKOV-3 cells (8×10^3 ^per well) were seeded in 96-well plates and cultured overnight. The next day, medium was replaced with fresh RPMI 1640 or medium containing chA21 (5 μg/ml), trastuzumab (5 μg/ml), or chA21 plus trastuzumab (5 + 5 μg/ml) for 12 h. After the supernatant was collected, the concentration of VEGF were measured using an ELISA kit for human VEGF (R&D Systems, USA) according to the manufacturer's instructions. The amount of VEGF in the supernatant was extrapolated from the VEGF standard curve and expressed in pg/ml. The levels of VEGF that could be detected in this assay ranged from 30-1200 pg/ml.

### HUVECs proliferation

SKOV-3 cells (4×10^3 ^per well) were seeded in 96-well plates. The next day cells were treated with chA21 (5 μg/ml), trastuzumab (5 μg/ml), or chA21 plus trastuzumab (5 + 5 μg/ml) for 48 h. The supernatant was collected and frozen at -20°C for the HUVECs proliferation assay. HUVECs were seeded in 96-well plates at a density of 5×10^3 ^per 100 μl and allowed to adhere overnight. Next, 100 μl of SKOV-3 supernatant was added to each well and HUVECs were cultured for 72 h. The number of HUVECs was measured by the MTS assay according to the manufactor's introduction (Promega, USA).

### HUVECs Migration assay

The HUVECs migration was assessed by Transwell assay (8 μm, Millipore, USA) in a double chamber co-culture system. Briefly, SKOV-3 cells (1.5×10^4^) were plated into 24-well plates (bottom chambers) and cultured with medium or medium supplemented with chA21 (5 μg/ml), trastuzumab (5 μg/ml), or chA21 (5 μg/ml) plus trastuzumab (5 μg/ml) for 24 h. HUVECs (8×10^3 ^per well) were seeded in Matrigel pre-coated Transwell chamber (top chamber), then the Transwell chambers were incubated into the 24-well plates. After co-cultured for 48 h, the top surfaces of the Transwell chambers were wiped with cotton swab. The migrated cells were fixed and stained with hematoxylin. Migration cells adhering to the undersurface of the filter were counted using an optical microscope (×400). Data was shown as the mean of the number of migrated HUVECs in five representative fields.

### Western blotting analysis

SKOV-3 cells were grown in 6-well dishes and treated with chA21 (5 μg/ml), trastuzumab (5 μg/ml), or both agents together (5 + 5 μg/ml) for 12 h. After the medium was removed, cells were washed twice with cold PBS and lysed in a 0.3 ml of radioimmunoprecipitation assay (RIPA) lysis buffer (20 mM sodium phosphate, pH 7.4, 150 mM NaCl, 1% Triton X-100, 5 mM EDTA, 5 mM phenylmethylsulfonyl fluoride, 10 mg/ml aprotinin, 10 mg/ml leupeptin, 250 mg/ml sodium vanadate) on ice. After removal of cell debris by centrifugation, protein concentration was determined by Lowry assay (Bio-Rad, USA). Cell lysates were subjected to 8% sodium dodecyl sulfate-polyacrylamide gel electrophoresis (SDS-PAGE) and then electrotransferred into the nitrocellulose membrane. After blocking with 5% defatted milk, the membrane was incubated separately with antibody against VEGF (1:500, Neomarkers), AKT (1:1000, Cell signaling Technology) or phospho-AKT (pAKT) at Ser473 (1:1000, Cell signaling Technology) for 2 h at room temperature. Sequently, the membrane was probed with horseradish peroxidase (HRP)-conjugated secondary goat anti-mouse antibody (1:10,000, Sigma) for 2 h at room temperature. Immunoreactive bands were developed with chemiluminescence (ECL) reagents (Pierce). The band were scanned for densitometric analysis using ImageJ 1.42 software (NIH, USA).

### Statistical analysis

Data are shown as means ± standard deviation (SD). Statistical analyses of the data were performed using one-way ANOVA test by SPSS 13.0. Value of *P *< 0.05 was considered statistically significant.

## Results

### Enhanced tumor growth inhibition by combination of chA21 with trastuzumab

**Figure 1:**
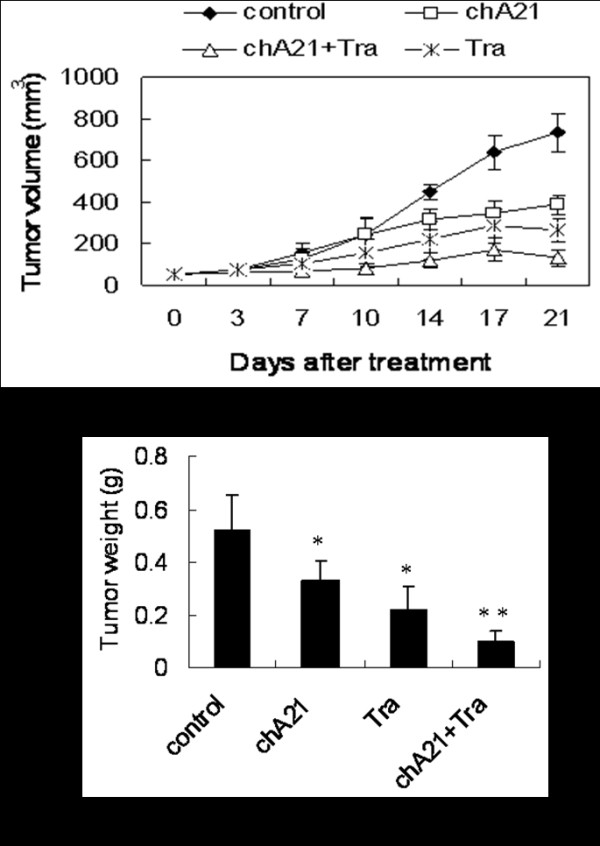
**The tumor volume and the weight of SKOV-3 xenograft in the different treatment groups**. (A) Either chA21 or trastuzumab treatment cause a marked growth inhibition in SKOV-3 xenograft compared with the control (*P *< 0.01), and the combination of chA21 with trastuzumab treatment induced a more efficient efficacy than the each antibody alone (*P *< 0.05). (B) When the experiment ended, all tumors were removed and weighted. Results are representative of the mean ± SD of 8 animals in each group. *, *P *< 0.01 compared with control. **, *P *< 0.01 compared with chA21 or trastuzumab alone.

Initially, we evaluated whether the chA21 plus trastuzumab treatment leads to better tumor inhibition in SKOV-3 xenografts. Female BALB/c nude mice were subcutaneously inoculated with human ovarian cancer cells SKOV-3 (5×10^6^) into the left flank of mice. Mice were randomized and injected twice weekly via i.v with either normal saline control, chA21 (30 mg/kg), trastuzumab (20 mg/kg), or chA21 plus trastuzumab (30 + 20 mg/kg) for 21 days. Either chA21 or trastuzumab alone treatment resulted in an effective suppression of tumor volume (Fig. 1A) and tumor weight (Fig. 1B) at day 21. The tumor inhibition ratios by chA21 or trastuzumab were 37% and 58%, respectively (*P *< 0.01). Moreover, the combination of chA21 and trastuzumab resulted in an 81% inhibition in tumor weight compared with the control (*P *< 0.001), which is greater than single treatment (*P *< 0.01). In addition, complete tumor eradication was seen in one mice from the combination treatment group.

### Increased anti-angiogenesis efficacy by combination of chA21 with trastuzumab

**Figure 2:**
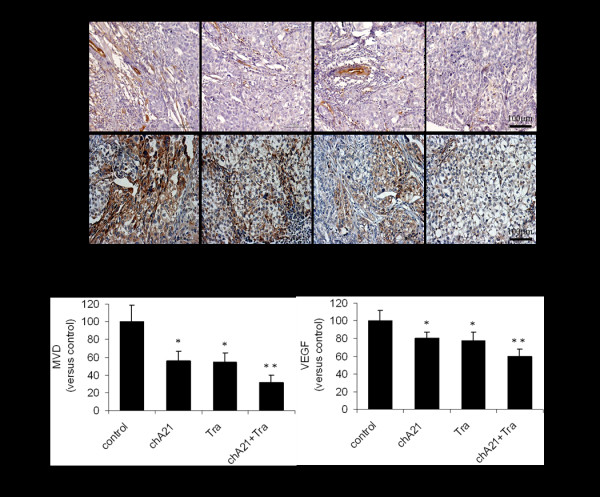
**Tumor microvessel density (MVD) and VEGF expression**. (A) The VEGF and CD34 (marker for MVD) expression in SKOV-3 xenografts were detected by immunohistochemistry. (B) Tumor MVD and MOD of VEGF expression were calculated and the values were shown as percents of the control treatment. *, *P *< 0.01 compared with control. **, *P *< 0.01 compared with chA21 or trastuzumab alone.

Angiogenesis plays an important role in cancer growth, we then examined whether the chA21 plus trastuzumab treatment leads to a more effective inhibition of angiogenesis than either treatment alone. MVD values were assessed by staining these with CD34 in tumor tissues that were removed from SKOV-3 xenografts. The most highly vascularized area of each tumor was identified on five high-powered fields were counted in this area of greatest vessel density. As shown in Fig. 2A and 2B, The number of MVD was 31% of the control in chA21 plus trastuzumab group, while this number was 56% in chA21 alone group and 54% in trastuzumab alone group. So chA21 combined with trastuzumab treatment resulted in a marked inhibition of tumor MVD compared with the control (*P *< 0.001) and either of chA21 or trastuzumab alone treatment (*P *< 0.01). Similarly, the tumor mean optical density (MOD) values of VEGF in the chA21 plus trastuzumab treatment group were 60% of the control, lower than those of 80% and 77% in individual treatment groups of chA21 and trastuzumab, respectively (*P *< 0.01).

### Augmented down-regulation of VEGF expression induced by combination of chA21 with trastuzumab

**Figure 3:**
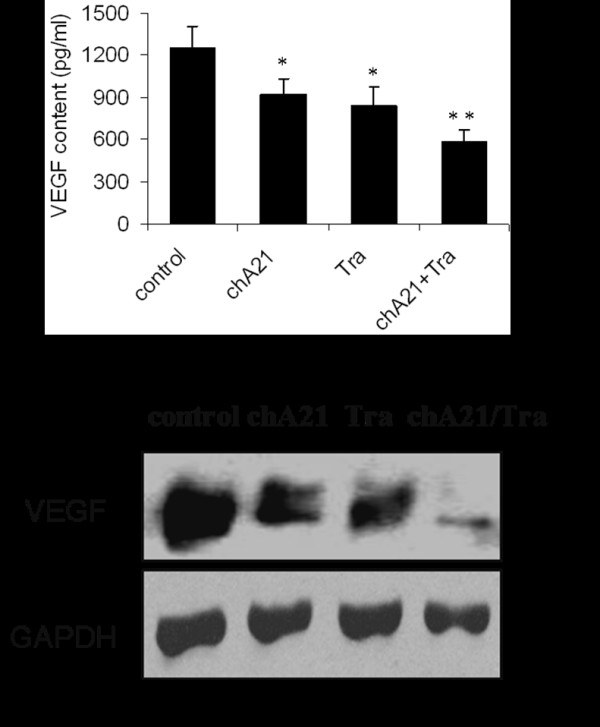
**Detection on secretion of VEGF from SKOV-3 cells**. (A) After co-culture of SKOV-3 cells with chA21 (5 μg/ml), trastuzumab (5 μg/ml) or chA21(5 μg/ml) plus trastuzumab (5 μg/ml) for 12 h, secreted content of VEGF in the medium was detected by ELISA. *, *P *< 0.01 compared with control. **, *P *< 0.01 compared with chA21 or trastuzumab alone. (B) VEGF protein expression in the SKOV-3 cells was detected by western blot.

Previouly, we found HER2 antibody inhibits angiogenesis and downregulats VEGF expression. Hence, we explored the effect of antibody synergy on VEGF seceration by ELISA test. We examined the amount of VEGF secreted into the medium from SKOV-3 cells that were treated with chA21, trastuzumab, or chA21 plus trastuzumab for 12 h. Compared with 1255.6 ± 153.6 pg/ml in the control group, the level of secreted VEGF decreased 918.7 ± 109.8 pg/ml in chA21 group (*P *< 0.01), 839.1 ± 137.8 pg/ml in trastuzumab group (*P *< 0.01), and 583.5 ± 87.7 pg/ml in the chA21 plus trastuzumab group (*P *< 0.001) (Fig. 3A).

We determined VEGF protein expression by Western blot upon HER2 antibody treatment for 12 h in SKOV-3 cells. In cosistent with ELISA data, we found treatment with chA21 or trastuzumab resulted in similar reduction of VEGF expression compared with the control. However, the combination of chA21 and trastuzumab induced a further inhibition of VEGF protein expression (Fig. 3B).

### Enhanced suppression of HUVECs proliferation and migration by combination of chA21 with trastuzumab

**Figure 4:**
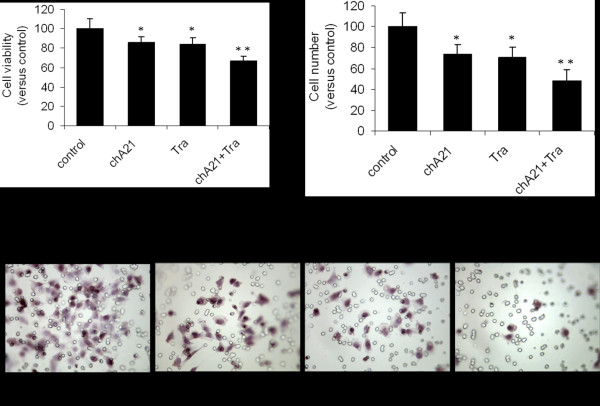
**HUVECs proliferation and migration assay**. (A) By the MTS assay, HUVECs number was measured by the MTS assay upon different treatment for 72 h. (B) The HUVECs migration was measured by Transwell assay in the co-cultured system (×400). (C) Data was shown as the mean of the number of migration HUVECs in five representative fields. *, *P *< 0.01 compared with control. **, *P *< 0.01 compared with chA21 or trastuzumab alone.

To further investigate the influence on the function of typical endothelial cells such as HUVECs, the effect of supernatant from SKOV-3 cells treated with antibodies on HUVECs proliferation was determined by the MTS assay. Compared with control group, HUVECs proliferation was inhibited by 33% in SKOV-3 supernatants treated with chA21 plus trastuzumab treatment group, 14% in chA21 group (*P *< 0.05) and 16% trastuzumab group (*P *< 0.05) (Fig. 4A).

HUVECs were co-cultured with SKOV-3 supernatants for 48 h. Migrated cells were stained and counted. As shown in Fig. 4B and 4C, the migrating capability of HUVECs was inhibited by SKOV-3 cell supernatant treated with chA21 plus trastuzumab compared with untreated cells (*P *< 0.001), chA21-treated cells (*P *< 0.01) or trastuzumab-treated cells (*P *< 0.01). The number of migrated cells (40×10 magnification) were 73%, 70% or 48% in chA21, trastuzumab, or combined treatment group compared with the control (Fig. 4C). Our data demonstrated that chA21 or trastuzumab treatment suppressed HUVECs migration and combination of chA21 and trastuzumab induced a further suppression.

### Potent inhibition of pAKT activity by combination of chA21 with trastuzumab

**Figure 5:**
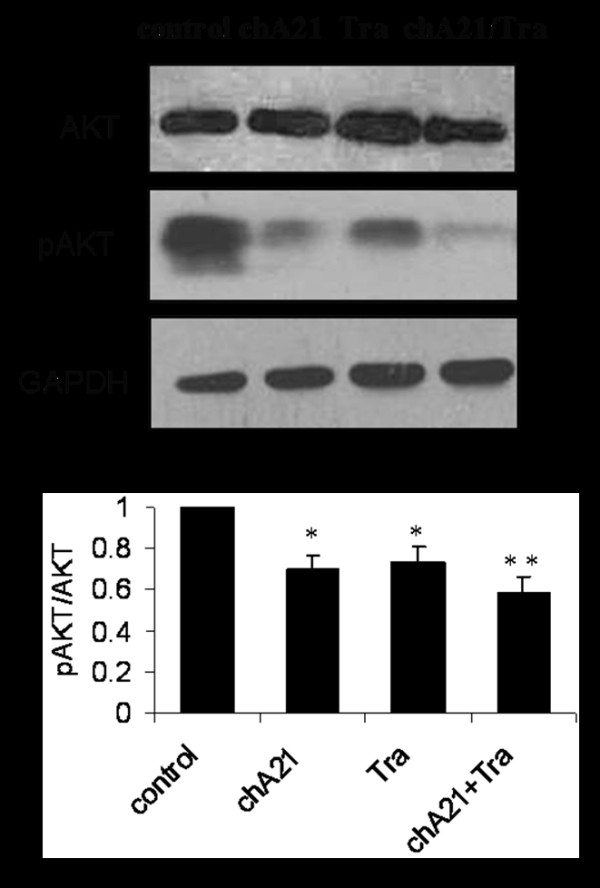
**Protein expression of AKT and phopsho-AKT (pAKT) in xenograft tumors**. (A) After SKOV-3 cells were treated with antibodies for 12 h, the cell lysates were analyzed. pAKT (Ser^473^) expression educed upon treament withchA21 or trastuzumab treatment and more dramatic reduction was observed with chA21 plus trastuzumab treatment. The levels of total AKT protein were not altered significantly upon various interventions. (B) The ratios of p-AKT/AKT were calculated and the values were shown as ratios compared with control, which was arbitrarily taken as 1.0. *, *P *< 0.01 compared with control. **, *P *< 0.01 compared with chA21 or trastuzumab alone.

To detect the underlying molecular mechanism of angiogenesis, we investigated the effect of chA21 plus trastuzumab treatment on AKT activity, which is a crucial pathway in angiogenesis [19]. After SKOV-3 cells were treated with antibodies for 12 h, the cell lysates were analyzed by Western blotting assay. As shown in Fig. 5A, pAKT (Ser^473^) expression were reduced by ChA21 or trastuzumab treatment, but more dramatic reduction was observed in the ChA21 plus trastuzumab treatment group. Total AKT protein were not altered significantly upon various interventions. As shown in Fig. 5B, The ratios of p-AKT/AKT were 0.61, 0.65 and 0.45 in chA21, trastuzumab and chA21 plus trastuzumab group. These data indicate that the ChA21 plus trastuzumab treatment cause greater inhibition of AKT expression compared with either treatment alone.

## Discussion

It's well known that HER2-overexpressing tumors confer enhanced metastasis-related properties and resistance to chemotherapeutic reagents which frequently result in poor clinical outcome. Trastuzumab as the first therapeutic anti-HER2 monoclonal antibody has been used in clinical treatment of HER2-overexpressing metastatic breast and gastric cancers [20,21]. It is also proposed to be a treatment option for patients with HER2-positive ovarian carcinoma [22]. However, poor responses and disease recurrences for trastuzumab therapy underscore for alternative treatments [23].

It is believed that angiogenesis is required for tumor growth and spread. HER2 signaling has been reported to be implicated in tumor angiogenesis [24,25]. Previously, we have developed a novel anti-HER2 chimeric antibody chA21 and this new antibody mainly binds to a distinct epitope on HER2 ECD I and inhibits tumor cells growth *in vitro *and *in vivo *[10,16]. In the present study we further explored the potential anti-angiogenic effects of chA21 on HER2-overexpressing ovarian carcinoma. Our data showed that chA21 and trastuzumab can inhibit tumor growth and angiogenesis in SKOV-3 xenograft model and the combination of trastuzumab with chA21 results in an enhanced effect.

Indeed, endothelial cells migration and proliferation is crucial for angiogenesis. Tumor cells induce angiogenesis by secreting various growth factors, such as VEGF, which binds its cognate receptor on endothelial cells and promotes these cells to proliferate and migrate [26,27]. Using ELISA kit to measuring the secreted VEGF from SKOV-3 cells, we found that chA21 could suppress VEGF expression and this synergism when combined chA21 with trastuzumab. Moreover, the synergism was confirmed by inhibition of HUVECs proliferation and migration when the endothelial cells were co-cultured with the supernatant from SKOV-3 cells treated with both trastuzumab and chA21 together. Therefore, the anti-angiogenesis capacity of the two antibodies alone and their synergy proved to be intrinsic.

Among signaling pathways induced by HER2 receptor, activation of the AKT kinase orchestrates a number of signaling pathways potentially involved in angiogenesis [19]. Our study revealed that chA21 could inhibit AKT expression with the capability similar to trastuzumab in SKOV-3 cell line. This result reflected the intrinsic properties of most tumor-inhibitory anti-HER2 antibodies to inhibit receptor-induced downstream signals at various efficiencies [28,29]. More importantly, the combination of chA21 with trastuzumab showed more significant potency on inhibiting the AKT. It may partly explain our findings that the combination of chA21 with trastuzumab could synergistically enhance the *in vitro *and *in vivo *anti-angiogenesis effects.

In conclusion, our study demonstrated the inhibition activities on tumor growth and angiogenesis of a novel anti-HER2 antibody chA21 alone and in combination with trastuzumab *in vitro *and *in vivo*. We found that the angiogenesis inhibition effect of chA21 could be enhanced by combination with trastuzumab, which might be mediated by synergism of chA21 and trastuzumab through inhibition of AKT expression. Therefore, chA21 may represent a unique anti-HER2 antibody with superior potentials as combination with other anti-HER2 reagents for further therapy.

## Competing interests

The authors declare that they have no competing interests.

## Authors' contributions

AZ and GS designed and conducted the studies, carried out corresponding data analyses, and drafted the manuscript. TZ participated in the animal experiments. GZ, JL, LS, WW, BL, ZW and QW participated in study design, coordination and helped to draft the manuscript. All authors have read and approved this final manuscript.
